# Denosumab ameliorates osteoarthritis by protecting cartilage against degradation and modulating subchondral bone remodeling

**DOI:** 10.1016/j.reth.2024.03.019

**Published:** 2024-03-26

**Authors:** Lei Shangguan, Ming Ding, Yingchun Wang, Hu Xu, Binghui Liao

**Affiliations:** Department of Orthopedic Surgery, Xijing Hospital, The Fourth Military Medical University, Xi'an, China

**Keywords:** Osteoarthritis, Denosumab, Osteoclast, Chondrocyte

## Abstract

Osteoarthritis (OA) is the most prevalent degenerative joint disease worldwide. Effective management for early-stage OA is crucial. Denosumab (DS) has been widely used to treat osteoporosis (OP) and rheumatoid arthritis, but its potential for managing OA remains clear. We assessed the effects of DS on osteoclast activity and chondrocyte apoptosis using tartrate-resistant acid phosphatase (TRAP) assay, quantitative real-time polymerase chain reaction (qRT-PCR), flow cytometry, and TUNEL staining. To assess the impact of DS on the NF-κB pathway, we performed Western blot and immunofluorescence staining. Additionally, we used an OA model to explore the influence of DS on subchondral bone remodeling and cartilage degeneration *in vivo*. We found that DS hindered receptor activator of nuclear factor kappa B ligand (RANKL)-induced osteoclastogenesis by inhibiting the activity of the NF-κB pathway. Besides, DS alleviated reactive oxygen species (ROS)-induced apoptosis in chondrocytes by regulating the expression of genes related to apoptosis. Moreover, we observed an attenuation of OA-related subchondral bone remodeling and cartilage degeneration *in vivo*. Our findings indicate that DS could effectively suppress osteoclast activity and chondrocyte apoptosis, thereby mitigating OA-related subchondral bone remodeling and cartilage degeneration. These results provide a mechanistic basis for using DS to treat OA.

## 1 Introduction

Osteoarthritis (OA) is a prevalent degenerative joint disease, with a significant impact on the patient's quality of life [1]. The clinical manifestations of OA include pain, joint stiffness, deformity, and advanced disability [2]. Current medications primarily alleviate pain rather than slow down or revert the progression of OA. It should be noted that end-stage OA patients often require joint replacement to restore joint function [3,4]. However, this surgery is financially costly and associated with potential complications. Therefore, there is an increasing demand for effective treatments for early-stage OA.

The main features of OA are subchondral bone remodeling and articular cartilage degeneration, suggesting abnormal activities of osteoclasts and chondrocytes, respectively [5]. Increased chondrocyte apoptosis in OA is strongly associated with the degradation of extracellular matrix components containing aggrecan and type II collagen. Several studies have shown that chondrocytes undergo apoptosis and trigger cartilage destruction in response to various stimuli, including metabolic and inflammatory factors [6,7]. However, previous research on the pathogenesis of OA has shown that osteoclast-mediated subchondral bone remodeling occurs prior to cartilage destruction during the progression of OA [8]. Furthermore, increased osteoclast activity and subchondral bone remodeling are important contributors to the thinning of the subchondral bone plate and bone loss in early-stage OA, which ultimately lead to articular cartilage catabolism [9,10]. Additionally, elevated secretion of transforming growth factor β (TGF-β) by osteoclasts in the subchondral bone at the onset of OA triggers apoptosis of chondrocytes, interrupting the integrity of extracellular matrix in the cartilage [11]. Therefore, interventions targeting osteoclastogenesis in subchondral bone may be an effective treatment for early-stage OA.

Denosumab (DS) is a human monoclonal antibody that specifically targets the receptor activator of nuclear factor kappa B ligand (RANKL), causing a reduction in both the activity and the survival of osteoclasts [12,13]. DS is a highly safe and effective agent for treating postmenopausal and metastatic cancer osteoporosis (OP), as well as inhibiting bone destruction in patients with rheumatoid arthritis (RA) [14,15]. OP and OA are two common degenerative diseases that are commonly found in older individuals [16]. Clinical observations have indicated a significant occurrence of both conditions among patients. Recent scientific investigations have uncovered remarkable similarities in the development and progression of OP and OA [17]. However, the precise roles of DS in treating OA remain unclear, calling for further research evaluate its efficacy.

This study characterizes the impact of DS on chondrocyte apoptosis and osteoclastogenesis, two representative cellular dysfunctions in OA. We found that DS could effectively inhibit osteoclast activity and chondrocyte apoptosis, hence alleviating OA-associated subchondral bone remodeling and cartilage degeneration, which provides a mechanistic basis for using DS to treat patients with early-stage OA.

## 2 Materials and methods

### 2.1 Cell culture

This study has been approved by the Ethics Committee of Xijing Hospital, Xi'an, China. All participating patients have signed the informed consent form. Human osteoclasts were isolated from peripheral blood mononuclear cells (PBMCs) *in vitro* as previously described [18]. Briefly, PBMCs were seeded onto a 96-well plate and then treated with macrophage colony-stimulating factor (M-CSF, 30 ng/mL, MedChemExpress, China) and RANKL (50 ng/Ml, MedChemExpress, China). Articular cartilage was extracted from patients undergoing total knee arthroplasty. The cartilage tissues were sectioned and then digested with type II collagenase (0.2%) at 37 °C for 12 h. Subsequently, chondrocytes were separated from the digested cartilage tissue and cultivated using DMEM with 10% fetal bovine serum (FBS) and 1% penicillin and streptomycin.

### 2.2 Cell proliferation and viability

We used the Cell Counting Kit-8 (CCK8) solution (Beyotime, Shanghai, China) and the 3-(4, 5) dimethylthiahiazo(-z-y1)-3,5-di-phenytetrazoliumromide (MTT) kit (Abmole, USA) to evaluate cell proliferation and viability, respectively. To assess cell proliferation, primary chondrocytes or PBMCs were seeded into 96-well plates with a density of 5000 cells/well and treated with DS (0.1, 1, 5, or 10 μM). After incubation for one day, 10 μL CCK-8 solution was added to each well and incubated for 60 min. We measured the absorbance at 450 nm using a microplate reader. For cell viability assessment, cells were treated with DS (0.1, 1, 5, or 10 μM) and cultured for 24 h. Cell viability was determined by MTT assay following the manufacturer's instructions.

### 2.3 Tartrate-resistant acid phosphatase (TRAP) staining

PBMCs were seeded onto a 96-well plate and then treated with M-CSF, RANKL (50 ng/mL), as well as DS (0 or 5 μM, MedChemExpress, China). After incubation for five days, the cells were fixed with 4% paraformaldehyde and stained with a TRAP working solution (Beyotime, Shanghai, China). Subsequently TRAP-positive osteoclasts were captured using microscopy (Carl Zeiss, Germany, 40x/NA 0.4).

### 2.4 Pit formation assay

PBMCs were cultured on bovine cortical bone slices and subsequently treated with M-CSF and RANKL (50 ng/mL) in the presence or absence of DS (0 or 5 μM). After incubation in osteoclastogenic medium, the bone slices were rinsed with 1 M ammonium chloride and subsequently stained with 0.5% toluidine blue.

### 2.5 Flow cytometry

An Annexin V-FITC/PI Apoptosis Kit (Beyotime, Shanghai, China) was used to identify the apoptosis rate of chondrocytes. After incubation for 24 h, chondrocytes were resuspended with binding buffer and stained with Annexin V-FITC/PI. Flow cytometry was used to examine and analyze various samples.

### 2.6 Quantitative real-time polymerase chain reaction (qRT-PCR)

To isolate total RNA, a TRIzol reagent (ThermoFisher, USA) was used. Subsequently, cDNA was synthesized using a reverse transcription kit (Takara, Dalian, China) and transcribed with a real-time PCR reagent (Takara, Dalian, China). Primer sequences are given in Supplementary Table 1.

### 2.7 Western blot

To extract total protein, RIPA lysis buffer was used. Subsequently, equal amounts of protein were obtained via SDS-PAGE and transferred onto polyvinylidene difluoride (PVDF) membranes. The membranes were blocked with skim milk and incubated with primary antibodies at 4 °C overnight. The membranes were subsequently incubated with horseradish peroxidase-conjugated secondary antibodies and visualized using Amersham Imager 600. Details of primary and secondary antibodies are given in Supplementary Table 2.

### 2.8 Immunofluorescence staining

Cells were fixed with 4% paraformaldehyde, permeabilized with 0.1% Triton X-100, and blocked with goat 5% serum. Subsequently, cells were incubated overnight at 4 °C with anti-IκBα (Cell Signaling Technology, #9242, 1:200) and anti-p65 (Cell Signaling Technology, #3034, 1:200) primary antibodies. The cells were then subjected to secondary antibody incubation, stained with DAPI, and visualized using fluorescence microscopy.

### 2.9 Reactive oxygen species (ROS) detection

The ROS levels in chondrocytes were determined using the ROS Assay Kit (Beyotime, Shanghai, China). Cells were incubated with DCFH-DA probes for 20 min at 37 °C and rinsed with culture medium three times. ROS-positive cells were identified and quantified using fluorescence microscopy.

### 2.10 TUNEL staining

Apoptotic chondrocytes were detected by the TUNEL method with the Cell Death Detection Kit (Roche, #11684809910, Switzerland). Briefly, the cells were incubated in the TUNEL solution, followed by staining with DAPI and imaging via fluorescence microscopy.

### 2.11 OA mouse model

Animal experiments were conducted in accordance with the protocols approved by the Animal Research Ethics Committee of the Fourth Military Medical University. The study utilized NOD-SCID mice from Cyagen Biosciences (C001316, China) to create humanized mice. Specifically, 5-week-old male NOD-SCID mice were irradiated with a sublethal dose (1.0 Gy) and then injected intravenously with 2 × 10^4^ human CD34^+^ monocytes. Subsequently, the NOD-SCID would develop a human immune system, including T cells and macrophages. To confirm the humanization process, peripheral blood samples were collected after five weeks to evaluate engraftment efficiency (the proportion of human CD34^+^ cells) using flow cytometry. Additionally, as the study focused on osteoclast activity in osteoarthritis, markers associated with macrophages (precursors of osteoclasts) were selected to verify the development of the human immune system in humanized mice.

OA was induced through surgical destabilization of the medial meniscus (DMM). The humanized mice were randomly assigned to one of the three groups: sham, DMM, and DMM + DS (*n* = 12 per group). In the sham group, the cranial meniscus tibial ligament was exposed without transection. In the DMM group, the cranial meniscus tibial ligament of the medial meniscus was transected to induce OA. In the DMM + DS group, the mouse underwent DMM surgery on left side and received DS by subcutaneous administration (10 mg/kg twice a week) for 4 weeks. Finally, the mice were sacrificed, and femur and tibia bones were collected for the subsequent studies.

### 2.12 Catwalk assessment

Catwalk analysis (CatWalk XTTM, Noldus, Netherlands) was used to evaluate pain-related behaviors in mice. Briefly, the mouse was placed on a glass plate illuminated by a fluorescent tube. Its footprints were captured by a high-speed camera in contact with the plate. Gait parameters including footprint area, mean intensity, stand time, swing time, and swing speed were recorded. The proportion of the left hindlimb to the right hindlimb (LH/RH) movement was also measured.

### 2.13 Micro-CT scanning and histological examination

The specimens underwent micro-CT scanning (eXplore Locus SP; GE Health Care Co., USA) and structural parameters, including bone mineral density (BMD), bone volume fraction (BV/TV), trabecular thickness (Tb.Th), trabecular number (Tb.N) and trabecular bone separation (Tb.Sp), were analyzed using Micview V2.1.2 3D reconstruction processing software. For histological examination, the specimens were fixed in 4% paraformaldehyde, decalcified in 10% EDTA, and embedded in paraffin. Subsequently, a range of histological examinations including hematoxylin and eosin (H&E), Safranin O, tartrate-resistant acid phosphatase (TRAP), as well as immunohistochemistry were conducted.

### 2.14 Statistical analysis

We used SPSS 13.0 for statistical analysis. Data are summarized as mean ± SD. We used unpaired two-tailed Student's *t*-test for comparisons between two groups, and one-way ANOVA followed by Bonferroni test for comparisons between more than two groups. P < 0.05 was considered significant.

## 3 Results

### 3.1 DS alleviated ROS-induced chondrocytes apoptosis

**Fig. 1:**
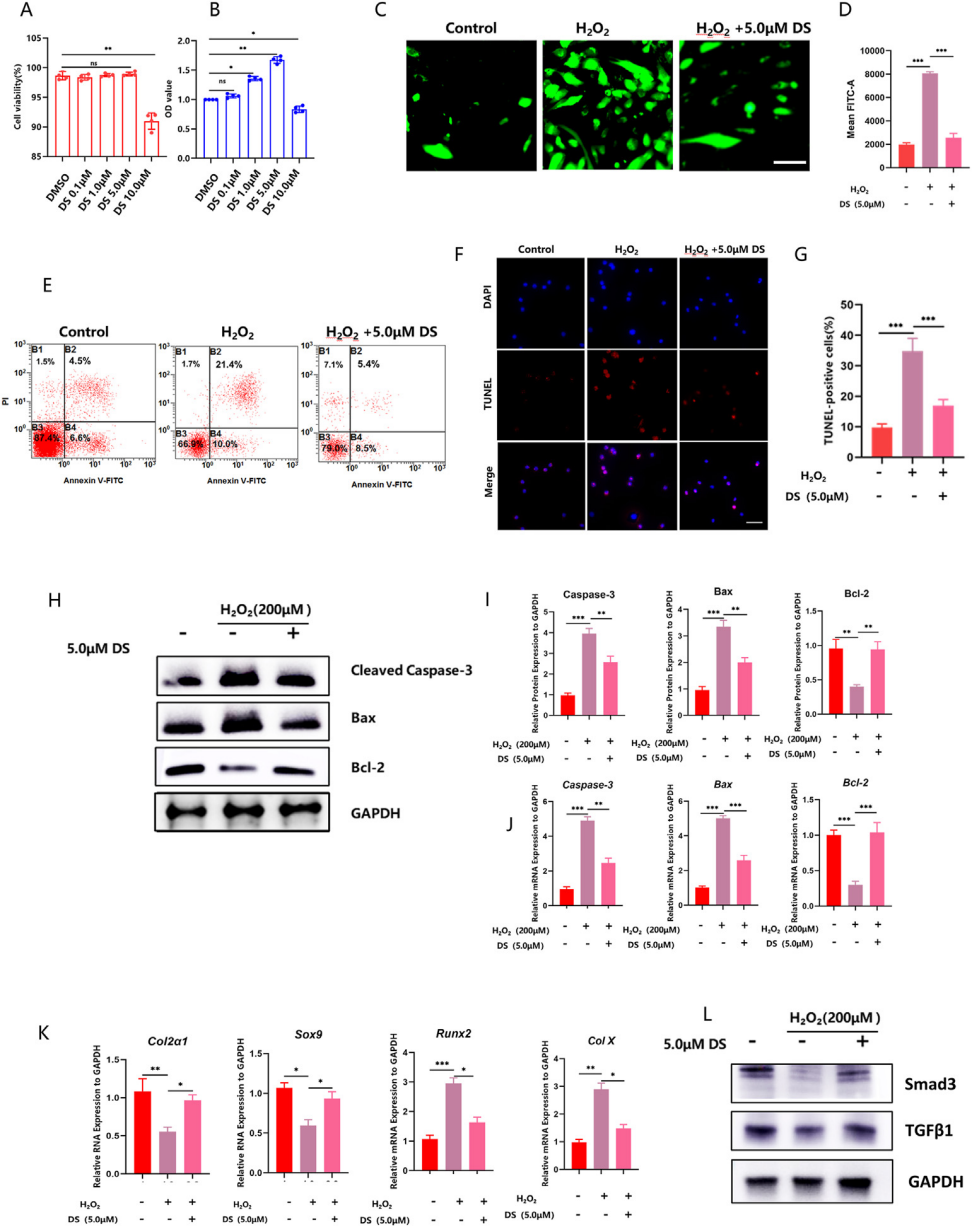
**DS alleviated ROS-induced chondrocytes apoptosis.** (A) The cytotoxic impact of DS at different concentrations, evaluated using an MTT assay. (B) The effect of different concentrations of DS on cell proliferation, determined by the CCK-8 assay. (C) Examples of immunofluorescence staining to measure intracellular ROS levels in cultured chondrocytes under different treatment conditions. (D) Semi-quantitative analysis of immunofluorescence intensity in cultured chondrocytes under different treatment conditions. (E) Flow cytometry results to assess cell apoptosis. (F, G) Examples of TUNEL staining (F) and quantification (G) to evaluate cell apoptosis. (H, I) Western blots to measure protein expression levels of apoptosis-related genes. (J) The mRNA levels of apoptosis-related genes. (K) The mRNA levels of SOX9, COL2A1, COL10A1 and RUNX2. (L) The proteins levels of TGFβ1 and smad3.

We first used the MTT assay to evaluate the cytotoxic effects of DS on chondrocytes. DS (concentration: 0.1 μM–5 μM) showed no cytotoxic effect on chondrocytes as compared to the DMSO control. However, the viability of chondrocytes significantly decreased after treatment with 10 μM DS (Fig. 1A). In addition, the CCK-8 assay demonstrated that 5 μM DS significantly increased chondrocyte proliferation, while 10 μM DS significantly inhibited chondrocyte proliferation (Fig. 1B). Thus, we selected 5 μM DS for further investigation. We subsequently evaluated the impact of DS on ROS-induced chondrocyte apoptosis through ROS detection and flow cytometry analysis. We found that DS effectively decreased ROS levels (Fig. 1C and D) and reduced the apoptosis rate of chondrocytes (Fig. 1E) induced by H_2_O_2_ treatment. The anti-apoptotic effect of DS on chondrocytes was also confirmed through TUNEL staining (Fig. 1F and G).

Furthermore, we evaluated the impact of DS on mRNA and protein expression of apoptosis-related genes through qRT-PCR and Western blot. QRT-PCR revealed that H_2_O_2_ treatment suppressed the expression of the anti-apoptotic gene bcl-2, while promoting the expression of the pro-apoptotic genes bax and caspase-3. However, the effect of H_2_O_2_ treatment was reversed by DS treatment (Fig. 1J). In addition, Western blot confirmed the anti-apoptotic effects of DS, showing that DS regulated the protein levels of bax, bcl-2, and cleaved caspase-3 (Fig. 1H and I). In addition, we investigated the impact of DS on chondrocyte differentiation. Through qRT-PCR analysis, we found that the expression of chondrocyte genes (*SOX9* and *COL2A1*) was suppressed by H_2_O_2_ treatment, while the expression of hypertrophy chondrocyte genes (*COLⅩ* and *RUNX2*) was promoted. However, the effects of H_2_O_2_ treatment were reversed by DS treatment (Fig. 1K). Previous research has suggested that the TGFβ1/smad3 signals inhibited hypertrophic differentiation of chondrocytes [19]. Therefore, we further examined the effects of DS on the TGFβ1/smad3 pathway in chondrocytes using Western blot analysis. Our results showed that DS treatment effectively inhibited hypertrophic differentiation of chondrocytes by upregulating the TGFβ1/smad3 pathway (Fig. 1L). These findings demonstrate that DS effectively reduces ROS-induced apoptosis hypertrophic differentiation in chondrocytes.

### 3.2 DS suppressed RANKL-induced osteoclastogenesis of peripheral blood mononuclear cells (PBMCs)

**Fig. 2:**
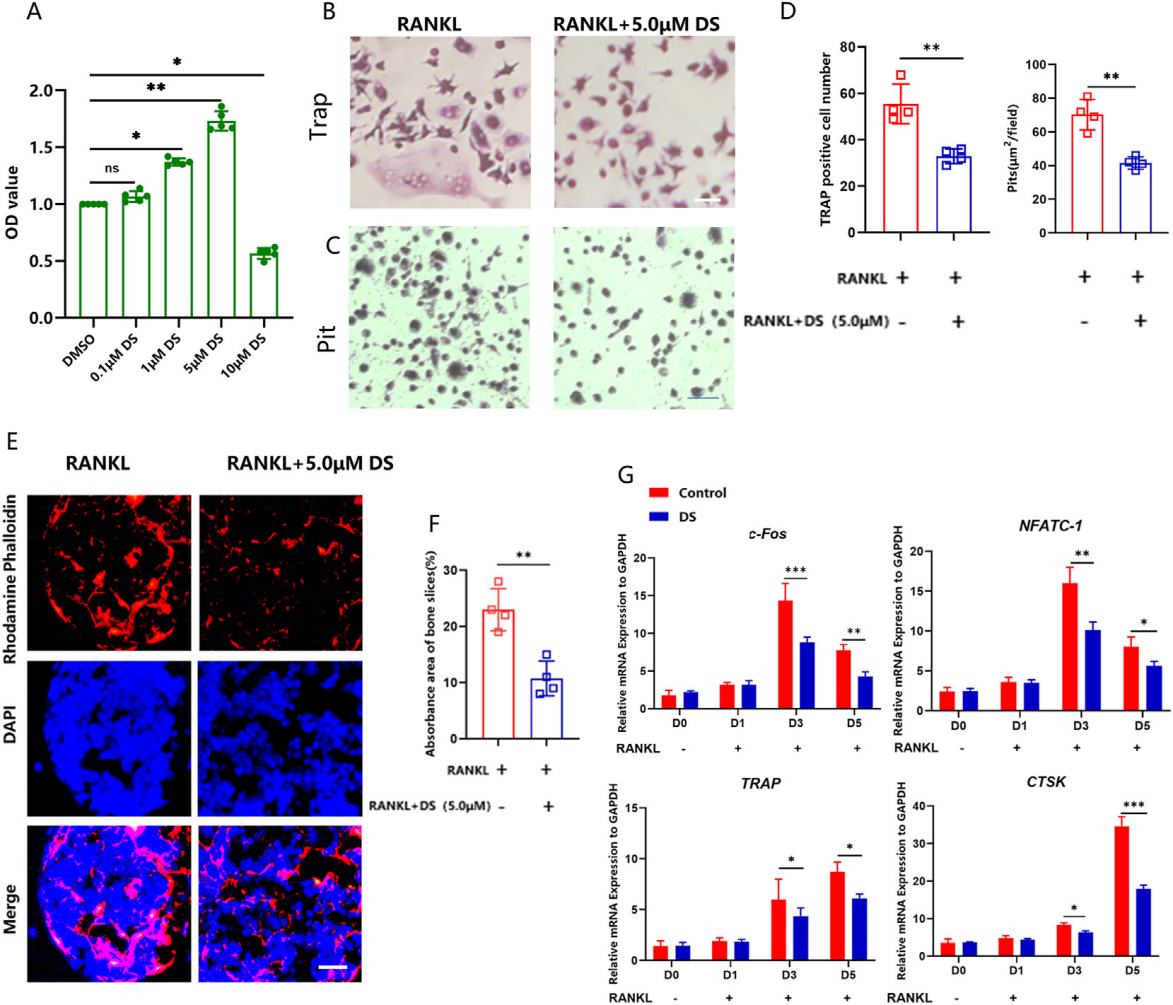
**DS suppressed RANKL-induced osteoclastogenesis of PBMCs.** (A) The effect of different concentrations of DS on cell proliferation, determined by the CCK-8 assay. (B, C, D) The effect of DS on osteoclast activity was assessed by TRAP staining and pit formation assay. (E, F) Examples of the F-actin ring in osteoclasts. (G) qRT-PCR measurement of the impact of DS on osteoclast-related gene expression.

To investigate the impact of DS on osteoclast activity, we stimulated PBMCs to differentiate into osteoclasts by introducing 50 ng/mL RANKL. Using the CCK-8 assay, we evaluated the cytotoxic impact of DS on chondrocytes. The CCK-8 assay demonstrated that 5 μM DS significantly increased osteoclast proliferation, while 10 μM DS significantly inhibited osteoclast proliferation (Fig. 2A). Based on these findings, it was evident that 5 μM DS was suitable for osteoclast assays. DS administration decreased the quantity of fully developed osteoclasts that tested positive for TRAP (Fig. 2B, D). In addition, there were fewer pit formations after DS treatment, suggesting a downregulation in osteoclast resorption capacity (Fig. 2C and D). We then performed immunofluorescence staining against the F-actin, as the F-actin ring in osteoclast cells serves as the primary structure for osteoclast bone resorption [20]; we found a significant reduction in both the size and the quantity of the F-actin ring after DS treatment (Fig. 2E and F). Next, we used qRT-PCR to evaluate the impact of DS on osteoclastogenesis. We focused on the transcription factors NFATc1 and c-FOS, as they are widely recognized to play a crucial role in regulating the expression of osteoclast-specific genes, including TRAP and CTSK [21]. We found that RANKL significantly enhanced the expressions of NFATc1 and c-FOS, as well as their target genes including TRAP and CTSK. Conversely, DS inhibited the up-regulation of these crucial transcription factors and osteoclastogenic genes induced by RANKL (Fig. 2G). These results suggest that DS has the potential to inhibit RANKL-induced osteoclastogenesis.

### 3.3 DS suppressed NF-κB pathway in RANKL-induced osteoclastogenesis

**Fig. 3:**
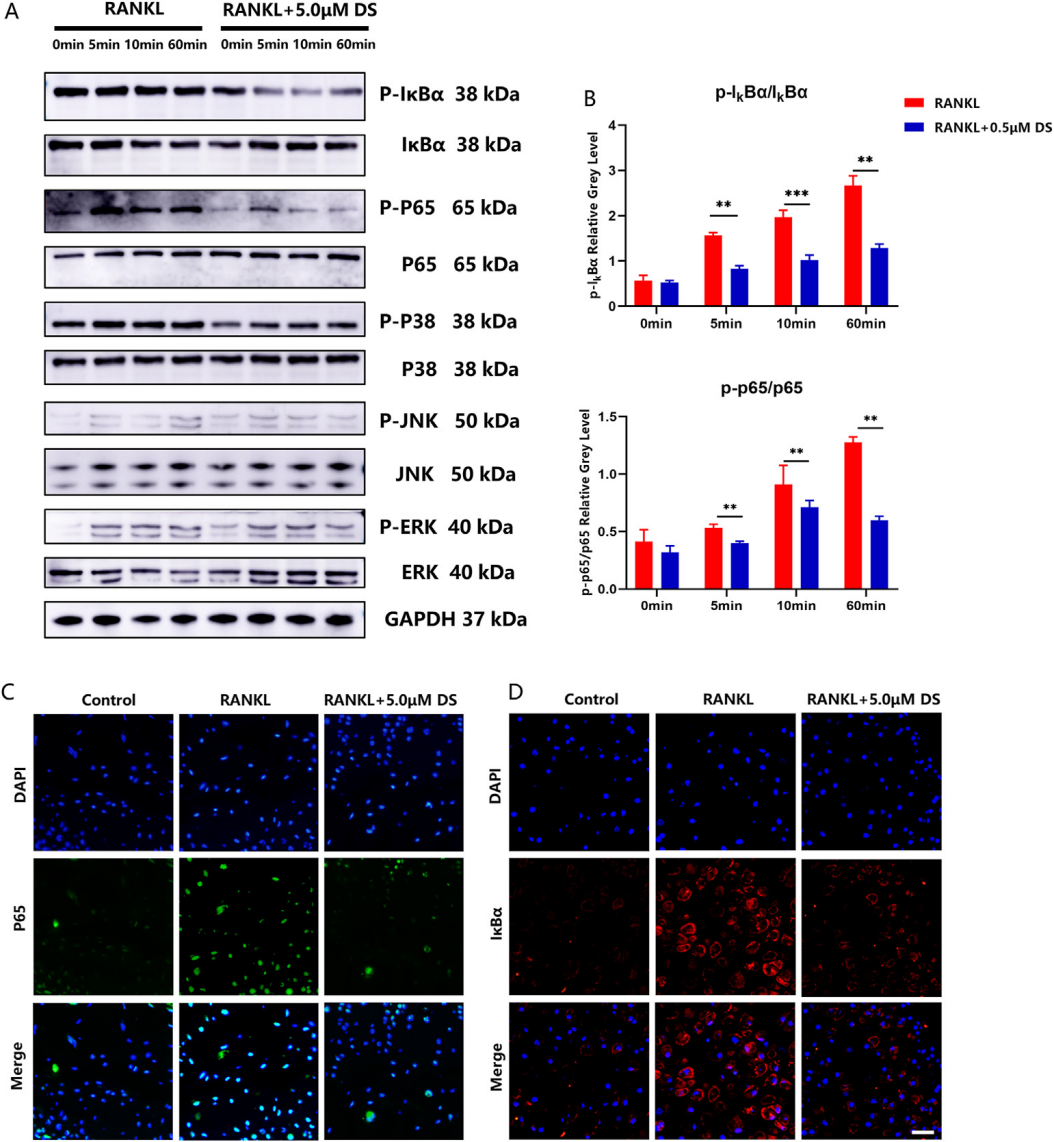
**DS suppressed the NF-κB pathway in RANKL-induced osteoclastogenesis.** (A) Western blot assessment of the activity of key proteins in the NF-κB pathway. (B) Quantification of IκBα and p65 activation as the ratio between phosphorylated and non-phosphorylated forms of proteins (p-IκBα/IκBα and p-p65/p65, respectively). (C, D) Examples of immunofluorescence staining of p65 and IκBα under different treatment conditions.

It is widely known that RANKL induces osteoclastogenesis through the NF-κB pathway [22]. We thus explored the effect of DS on the NF-κB pathway in PBMCs using Western blot and immunofluorescence staining. Western blot revealed that DS treatment effectively reversed the increased phosphorylation of IκBα and p65 (Fig. 3A and B). In addition, DS treatment had an impact on the phosphorylation of p38 (Supplementary Fig. 1). However, it was observed that the phosphorylation of IκBα and p65 was more pronounced compared to the phosphorylation of p38. Therefore, we decided to focus our further investigation on IκBα and p65. This effect was further confirmed by immunofluorescence staining of p65 and IκBα (Fig. 3C and D). These findings strongly suggest that DS can suppress RANKL-induced osteoclastogenesis by inhibiting the NF-κB signaling pathway.

### 3.4 DS treatment ameliorated pain-related behaviors in mice

Flow cytometry analysis and immunofluorescence were performed to confirm the humanization process, Flow cytometry analysis revealed an engraftment efficiency of 60% (Supplementary Fig. 2A), considered successful as previous studies suggested an efficiency above 10% indicated successful modeling [23]. Immunofluorescence results indicated a higher proportion of CD14 positive cells in the spleen of humanized mice compared to non-humanized mice (Supplementary Fig. 2B), demonstrating the development of a humanized immune system in the mice.

**Fig. 4:**
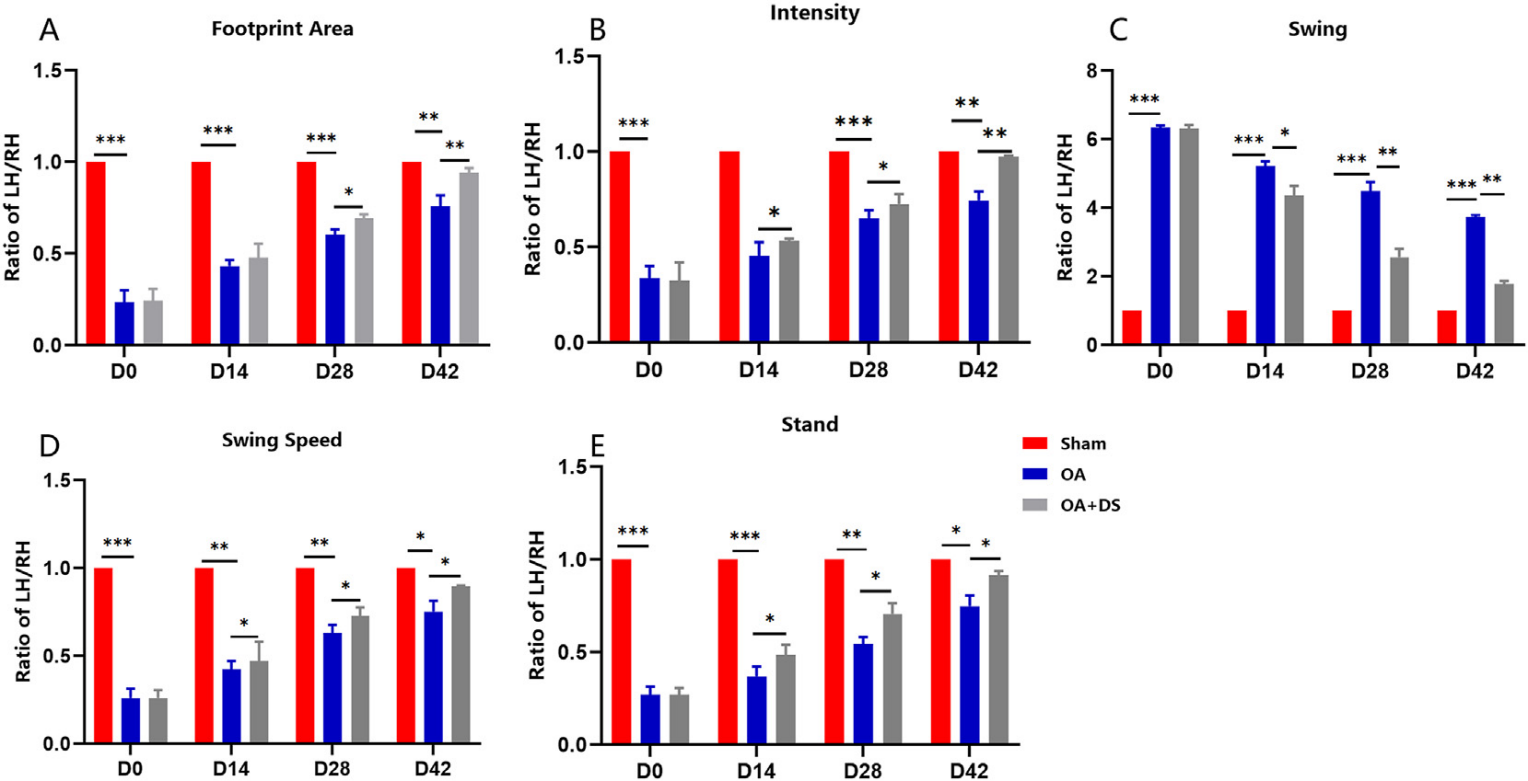
**DS treatment ameliorated pain-related behavior in mice.** (A–E) Quantification gait parameters, including footprint area (A), mean intensity (B), swing time (C), swing speed (D), and increased stand time (E) in different groups over time.

As pain is a prominent symptom in OA patients, we also assessed the impact of DS treatment on pain-related behaviors in the mouse DMM model of OA. To evaluate pain, we used the widely recognized catwalk analysis. The catwalk analysis evaluates the ratio between the measurements of left and right hindlimbs. The baseline inter-limb coordination was measured using the regularity index, which is the ratio of the left hindlimb to the right hindlimb. The analysis demonstrated that DS treatment ameliorated pain-related behaviors in mice, shown as increased footprint area (Fig. 4A), increased intensity (Fig. 4B), reduced swing (Fig. 4C), increased swing speed (Fig. 4D) and increased stand time (Fig. 4E).

### 3.5 DS treatment ameliorated OA progression *in vivo*

**Fig. 5:**
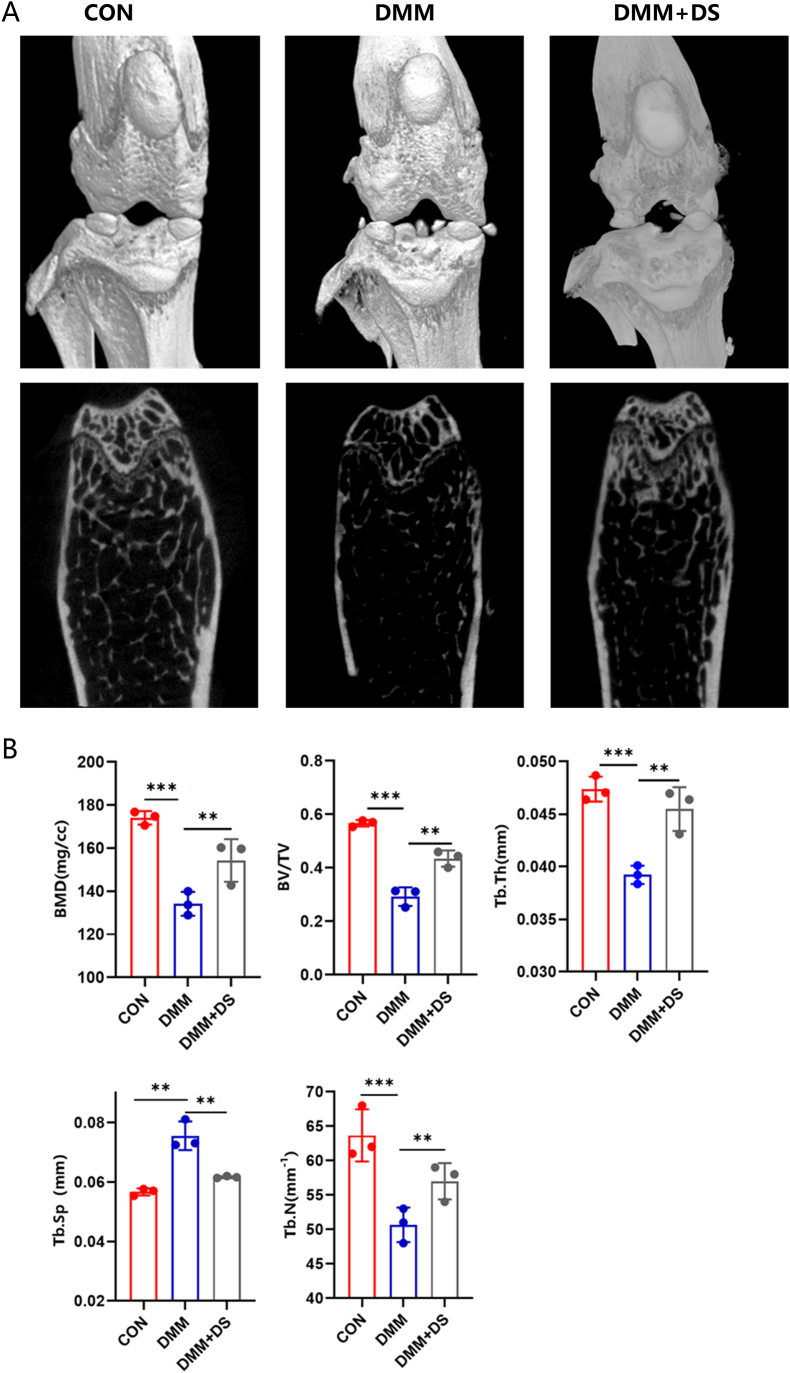
**DS treatment inhibited abnormal subchondral bone remodeling.** (A) Examples of 3D micro-CT images of the knee joint in mice receiving sham surgery, DMM, and DMM + DS treatment. (B) Quantitative analysis of structural parameters, including BMD, BV/TV, Tb.Th, Tb.N, and Tb.Sp. in mice receiving sham surgery, DMM, and DMM + DS treatment.

**Fig. 6:**
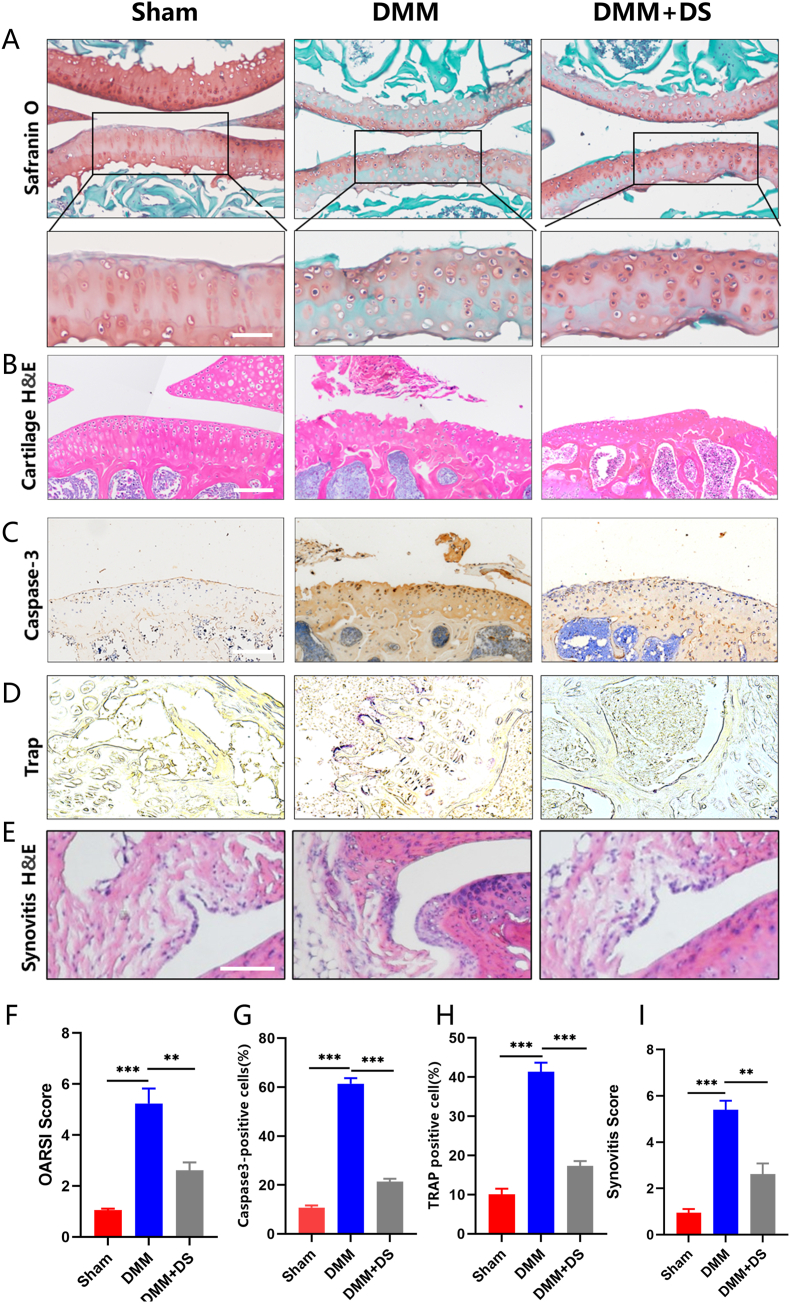
**DS treatment ameliorated OA progression *in vivo*.** (A) Examples of Safranin O staining. (B) Examples of H&E staining. (C) Examples of immunohistochemistry of caspase-3. (D) Examples of TRAP staining. (E) HE staining of synovitis. (F) The Osteoarthritis Research Society International (OARSI) score to assess cartilage degeneration in mice receiving sham surgery, DMM, and DMM + DS treatment. (G) Percentage of cells that were caspase-3^+^ in mice receiving sham surgery, DMM, and DMM + DS treatment. (H) Percentage of cells that were TRAP^+^ in mice receiving sham surgery, DMM, and DMM + DS treatment. (I) The quantification of synovitis scores.

We then evaluated the therapeutic effects of DS on OA progression *in vivo* in the mouse DMM model. We conducted micro-CT analysis to evaluate subchondral bone remodeling, and found that DMM caused significant subchondral bone resorption. However, this detrimental effect was effectively reversed by DS treatment (Fig. 5A). The results were further confirmed by quantitative analysis of structural parameters, including BMD, BV/TV, Tb.Th, Tb.N, and Tb.Sp (Fig. 5B). Also, H&E and Safranin O/Fast Green staining demonstrated that the destroyed articular cartilage and reduced proteoglycan in the DMM group were restored by DS treatment (Fig. 6A, B, F). TRAP staining displayed early hyperactivity of the osteoclasts in the DMM group, which was inhibited after DS treatment (Fig. 6D, H). Furthermore, caspase-3 staining showed that DS attenuated chondrocyte apoptosis in degenerated cartilage induced by DMM surgery (Fig. 6C, G). In order to evaluate the effect of DS on synovitis, three features of synovitis were assessed: the enlargement of the lining cell layer, cellular density of synovial stroma, and leukocytic infiltrate [24]. The results showed that DS reduced these features, indicating its impact on the synovium (Fig. 6E). This finding was further supported by the synovitis scores (Fig. 6I). In summary, DS treatment ameliorated OA progression *in vivo*.

## 4 Discussion

The primary objectives of treating osteoarthritis (OA) are to alleviate pain, enhance functionality, and improve the overall quality of life [25]. Key features of OA include subchondral bone remodeling and degeneration of articular cartilage [26]. Recent research has shown that in early-stage OA, subchondral bone remodeling occurs before cartilage degeneration, indicating its importance in both the development and the progression of OA [27]. In addition, macrophage infiltration and osteoclast activation play an important role in subchondral bone remodeling during early-stage OA [28].

Recent studies have indicated that certain OP treatments may also have therapeutic effects on OA [29]. Zoledronic acid (zoledronate) is one of the preferred drugs for OP treatment [30]. Animal experiments focusing on OA have shown that zoledronate exhibits a partial effect on cartilage proteins, thereby enhancing the local osteochondral barrier and maintaining the thickness of subchondral and cancellous bones [31]. These findings hold significance for the treatment of OA. However, it is important to note that zoledronic acid requires intravenous injection on an annual basis, which may lead to side effects in some patients after infusion [32]. Parathyroid hormone is a novel bone formation promoter that specifically binds to cell membrane protein-coupled receptors [33]. It activates adenylate cyclase and phospholipase, leading to an increase in the number and activity of osteoblasts, thereby enhancing new bone formation [34]. The FDA has approved teriparatide for the treatment of osteoporosis in postmenopausal women, aiming to reduce the risk of fractures [35]. Additionally, Liang et al. found that teriparatide could potentially inhibit the overexpression of MMP-13 induced by TNF-α. This inhibition might contribute to the prevention of synovitis and cartilage degradation, while simultaneously promoting the regeneration of articular cartilage [36]. However, teriparatide injections are quite costly and need to be administered daily [37]. In comparison, DS is a fully humanized monoclonal antibody that targets the receptor activator of nuclear factor kappa-B ligand (RANKL). It is injected subcutaneously only once during the first half of the year for the treatment of OP [38]. This approach is more efficient and convenient. Our study found that DS was extremely effective in treating OA by inhibiting the activity and survival of osteoclasts.

Osteoclast hyperactivity is widely recognized as a significant characteristic of bone resorption [39]. RANKL is a factor responsible for the differentiation of osteoclasts and facilitates their formation and activation [40]. The interaction between RANKL and its receptor RANK is of utmost importance in osteoclast formation and activation [41]. In this study, we induced PBMCs to differentiate into osteoclast cells by adding M-CSF and RANKL to assess the effects of DS on osteoclast activity. Our findings demonstrated that DS hindered the RANKL-induced osteoclastogenesis of PBMCs. Subsequently, we investigated the underlying mechanism through which DS influenced RANKL-induced osteoclastogenesis. The results of Western blot and immunofluorescence staining confirmed that DS suppressed osteoclastogenesis induced by RANKL by down-regulating the expression of osteoclast-specific genes, thereby blocking the NF-κB signaling pathway. Finally, the OA model was utilized to evaluate the effect of DS on subchondral bone remodeling. The findings demonstrated that DS restored the bone resorption of subchondral bone induced by DMM surgery via inhibiting osteoclast hyperactivity, verified by micro-CT analysis and TRAP staining. Bone remodeling is the primary mode of bone turnover, in which resorption and formation are normally balanced. In the presence of the RANKL inhibitor DS, osteoclast activation is inhibited, which ameliorates subchondral bone loss.

Chondrocytes play a crucial role in the progression of articular cartilage degeneration in OA patients [42]. Chondrocyte apoptosis leads to the loss of extracellular matrix (ECM) components, including aggrecan and type II collagen [43]. ECM components contribute to the tensile strength and compressive elasticity of the joint, supporting its fundamental functions [43]. However, ECM loss can precipitate the progression of OA [44]. In addition, ROS-induced chondrocyte apoptosis is a crucial event in OA [42]. ROS are chemical byproducts generated during the mitochondrial electron transport chain. They serve as important intermediates in multiple signaling pathways, including apoptosis [45]. In our study, DS treatment inhibited chondrocyte apoptosis induced by H_2_O_2_ *in vitro*. Furthermore, histological examination revealed that DS treatment restored the destroyed articular cartilage. The results obtained from both Safranin O/Fast Green staining and H&E staining were consistent and comparable. Therefore, our findings suggested that DS protects cartilage from degeneration by preventing chondrocyte apoptosis induced by oxidative stress.

In conclusion, our research has demonstrated that DS treatment effectively suppressed osteoclast activation by inhibiting the NF-κB pathway and chondrocyte apoptosis induced by H_2_O_2_. These dual effects ultimately attenuated OA-related subchondral bone remodeling and cartilage degeneration. As OP and OA share certain pathophysiological and epidemiological features, our findings provide a mechanistic basis for repurposing an effective OP drug as a therapeutic agent for early-stage OA. Future research is needed to clarify the relationship between OP and OA, and to elucidate the impact of DS on other signaling pathways implicated in OA.

## Ethics approval and consent to participate

The study was approved by the Ethics Committee of The Fourth Military Medical University.

## Declaration of competing interest

The authors declare there are no competing interest.

## References

[1] Arnold J.B., Bowen C.J., Chapman L.S., Gates L.S., Golightly Y.M., Halstead J., Hannan M.T., Menz H.B., Munteanu S.E., Paterson K.L. (2022). International Foot and Ankle Osteoarthritis Consortium review and research agenda for diagnosis, epidemiology, burden, outcome assessment and treatment. Osteoarthritis Cartilage.

[2] Litwic A., Edwards M.H., Dennison E.M., Cooper C. (2013). Epidemiology and burden of osteoarthritis. Br Med Bull.

[3] Arden N.K., Perry T.A., Bannuru R.R., Bruyère O., Cooper C., Haugen I.K., Hochberg M.C., McAlindon T.E., Mobasheri A., Reginster J.Y. (2021). Non-surgical management of knee osteoarthritis: comparison of ESCEO and OARSI 2019 guidelines. Nat Rev Rheumatol.

[4] Allen K.D., Thoma L.M., Golightly Y.M. (2022). Epidemiology of osteoarthritis. Osteoarthritis Cartilage.

[5] Hunter D.J., Bierma-Zeinstra S. (2019). Osteoarthritis. Lancet (London, England).

[6] Lepetsos P., Papavassiliou A.G. (2016). ROS/oxidative stress signaling in osteoarthritis. Biochim Biophys Acta.

[7] Bolduc J.A., Collins J.A., Loeser R.F. (2019). Reactive oxygen species, aging and articular cartilage homeostasis. Free Radic Biol Med.

[8] Meachim G., Ghadially F.N., Collins D.H. (1965). Regressive changes in the superficial layer of human articular cartilage. Ann Rheum Dis.

[9] Goldring S.R. (2009). Role of bone in osteoarthritis pathogenesis. Med Clin.

[10] Goldring M.B., Goldring S.R. (2010). Articular cartilage and subchondral bone in the pathogenesis of osteoarthritis. Ann N Y Acad Sci.

[11] Hu W., Chen Y., Dou C., Dong S. (2021). Microenvironment in subchondral bone: predominant regulator for the treatment of osteoarthritis. Ann Rheum Dis.

[12] Hu Q., Zhong X., Tian H., Liao P. (2021). The efficacy of denosumab in patients with rheumatoid arthritis: a systematic review and pooled analysis of randomized or matched data. Front Immunol.

[13] Tanaka S., Tanaka Y. (2021). RANKL as a therapeutic target of rheumatoid arthritis. J Bone Miner Metabol.

[14] Kostenuik P.J., Nguyen H.Q., McCabe J., Warmington K.S., Kurahara C., Sun N., Chen C., Li L., Cattley R.C., Van G. (2009). Denosumab, a fully human monoclonal antibody to RANKL, inhibits bone resorption and increases BMD in knock-in mice that express chimeric (murine/human) RANKL. J Bone Miner Res.

[15] Fuksa L., Vytrisalova M. (2015). Adherence to denosumab in the treatment of osteoporosis and its utilization in the Czech Republic. Curr Med Res Opin.

[16] Lin L., Luo P., Yang M., Wang J., Hou W., Xu P. (2022). Causal relationship between osteoporosis and osteoarthritis: a two-sample Mendelian randomized study. Front Endocrinol.

[17] Stamenkovic B.N., Rancic N.K., Bojanovic M.R., Stojanovic S.K., Zivkovic V.G., Djordjevic D.B., Stankovic A.M. (2022). Is osteoarthritis always associated with low bone mineral density in elderly patients?. Medicina.

[18] Zhang M., Huang B. (2012). The multi-differentiation potential of peripheral blood mononuclear cells. Stem Cell Res Ther.

[19] Rim Y.A., Nam Y., Ju J.H. (2020). The role of chondrocyte hypertrophy and senescence in osteoarthritis initiation and progression. Int J Mol Sci.

[20] Matsubara T., Kinbara M., Maeda T., Yoshizawa M., Kokabu S., Takano Yamamoto T. (2017). Regulation of osteoclast differentiation and actin ring formation by the cytolinker protein plectin. Biochem Biophys Res Commun.

[21] Kim K., Kim T.H., Ihn H.J., Kim J.E., Choi J.Y., Shin H.I., Park E.K. (2018). Inhibitory effect of purpurogallin on osteoclast differentiation in vitro through the downregulation of c-fos and NFATc1. Int J Mol Sci.

[22] Tao H., Li W., Zhang W., Yang C., Zhang C., Liang X., Yin J., Bai J., Ge G., Zhang H. (2021). Urolithin A suppresses RANKL-induced osteoclastogenesis and postmenopausal osteoporosis by, suppresses inflammation and downstream NF-κB activated pyroptosis pathways. Pharmacol Res.

[23] Bodic B., Boutet M.A., Boyer C., Metayer B., Vignes C., Lesoeur J., Veziers J., Daguin V., Haspot F., Maugars Y. (2022). Development and characterization of a humanized mouse model of osteoarthritis. Osteoarthritis Cartilage.

[24] Krenn V., Morawietz L., Burmester G.R., Kinne R.W., Mueller-Ladner U., Muller B., Haupl T. (2006). Synovitis score: discrimination between chronic low-grade and high-grade synovitis. Histopathology.

[25] Tsokanos A., Livieratou E., Billis E., Tsekoura M., Tatsios P., Tsepis E., Fousekis K. (2021). The efficacy of manual therapy in patients with knee osteoarthritis: a systematic review. Medicina.

[26] Englund M., Roemer F.W., Hayashi D., Crema M.D., Guermazi A. (2012). Meniscus pathology, osteoarthritis and the treatment controversy. Nat Rev Rheumatol.

[27] Lin C., Chen Z., Guo D., Zhou L., Lin S., Li C., Li S., Wang X., Lin B., Ding Y. (2022). Increased expression of osteopontin in subchondral bone promotes bone turnover and remodeling, and accelerates the progression of OA in a mouse model. Aging (Albany NY).

[28] Zhou F., Mei J., Han X., Li H., Yang S., Wang M., Chu L., Qiao H., Tang T. (2019). Kinsenoside attenuates osteoarthritis by repolarizing macrophages through inactivating NF-κB/MAPK signaling and protecting chondrocytes. Acta Pharm Sin B.

[29] Xin Y., Tang A., Pan S., Zhang J. (2021). Components of the endocannabinoid system and effects of cannabinoids against bone diseases: a mini-review. Front Pharmacol.

[30] Recknor C. (2011). Zoledronic acid for prevention and treatment of osteoporosis. Expet Opin Pharmacother.

[31] Xu L., Hu Y.J., Peng Y., Wang Z., Wang J., Lu W.W., Tang B., Guo X.E. (2023). Early zoledronate treatment inhibits subchondral bone microstructural changes in skeletally-mature, ACL-transected canine knees. Bone.

[32] Reid I.R., Green J.R., Lyles K.W., Reid D.M., Trechsel U., Hosking D.J., Black D.M., Cummings S.R., Russell R.G.G., Eriksen E.F. (2020). Zoledronate. Bone.

[33] Rendina-Ruedy E., Rosen C.J. (2022). Parathyroid hormone (PTH) regulation of metabolic homeostasis: an old dog teaches us new tricks. Mol Metabol.

[34] Chen T., Wang Y., Hao Z., Hu Y., Li J. (2021). Parathyroid hormone and its related peptides in bone metabolism. Biochem Pharmacol.

[35] Lindsay R., Krege J.H., Marin F., Jin L., Stepan J.J. (2016). Teriparatide for osteoporosis: importance of the full course. Osteoporos Int.

[36] Liang X., Li S.R., Zhang X.X., He S.H., Li S.S., Li T.F. (2023). Teriparatide prevented synovial inflammation and cartilage destruction in mice with DMM. Connect Tissue Res.

[37] Saag K.G., Shane E., Boonen S., Marín F., Donley D.W., Taylor K.A., Dalsky G.P., Marcus R. (2007). Teriparatide or alendronate in glucocorticoid-induced osteoporosis. N Engl J Med.

[38] Kobayakawa T., Miyazaki A., Saito M., Suzuki T., Takahashi J., Nakamura Y. (2021). Denosumab versus romosozumab for postmenopausal osteoporosis treatment. Sci Rep.

[39] Kim H., Lee K., Kim J.M., Kim M.Y., Kim J.R., Lee H.W., Chung Y.W., Shin H.I., Kim T., Park E.S. (2021). Selenoprotein W ensures physiological bone remodeling by preventing hyperactivity of osteoclasts. Nat Commun.

[40] Bonnet N., Bourgoin L., Biver E., Douni E., Ferrari S. (2019). RANKL inhibition improves muscle strength and insulin sensitivity and restores bone mass. J Clin Invest.

[41] Udagawa N., Koide M., Nakamura M., Nakamichi Y., Yamashita T., Uehara S., Kobayashi Y., Furuya Y., Yasuda H., Fukuda C. (2021). Osteoclast differentiation by RANKL and OPG signaling pathways. J Bone Miner Metabol.

[42] Wang B.W., Jiang Y., Yao Z.L., Chen P.S., Yu B., Wang S.N. (2019). Aucubin protects chondrocytes against IL-1β-induced apoptosis in vitro and inhibits osteoarthritis in mice model. Drug Des Dev Ther.

[43] Hwang H.S., Kim H.A. (2015). Chondrocyte apoptosis in the pathogenesis of osteoarthritis. Int J Mol Sci.

[44] Guo Q., Chen X., Chen J., Zheng G., Xie C., Wu H., Miao Z., Lin Y., Wang X., Gao W. (2021). STING promotes senescence, apoptosis, and extracellular matrix degradation in osteoarthritis via the NF-κB signaling pathway. Cell Death Dis.

[45] Musumeci G., Castrogiovanni P., Trovato F.M., Weinberg A.M., Al-Wasiyah M.K., Alqahtani M.H., Mobasheri A. (2015). Biomarkers of chondrocyte apoptosis and autophagy in osteoarthritis. Int J Mol Sci.

