# Supplementary material for: Denosumab ameliorates osteoarthritis by protecting cartilage against degradation and modulating subchondral bone remodeling
Source: Regen Ther. 2024 Mar 26;27:181–90. doi: 10.1016/j.reth.2024.03.019 (PMC11150975; doi:10.1016/j.reth.2024.03.019)
Supplement: Multimedia component 1 [file mmc1.docx]

**Supplementary table 1: The premiers used in this study.**

| **Gene** | **Primer sequences (5’-3’)** |
| --- | --- |
| GAPDH (F) | AGCTACTCGCGGCTTTACG |
| GAPDH (R) | ATCCGTTCACACCGACCTTC |
| Caspase-3 (F) | AAGAGCAGCATGACCTCTCAC |
| Caspase-3 (R) | CTGCCTGAAATCATTGCCT |
| Bax (F) | CGACCGACAAGGAGCTAGAGG |
| Bax (R) | CCGTGACAGGCTTGGCACTA |
| Bcl2 (F) | GTGTATGACCAATACTTGCCAC |
| Bcl2 (R) | ACTGCCACTGTTGGAGAC |
| Fos (F) | ATGTTGCCGCATCTGTGTGT |
| Fos (R) | CTGCTTTCTGCCCCTTTGGC |
| NFATC-1 (F) | GGCTACCGGGTACCCTACAG |
| NFATC-1 (R) | AGATTGCTCCTGGTCTGCAA |
| TRAP (F) | GTGTATGAGCAATACTTGGCAC |
| TRAP (R) | ACTGCCTCTGTTGGTGAC |
| CTSK (F) | ATGAGCAGCATGAGCTCTCAC |
| CTSK (R) | CAGCCTGAAATCTTTGCCT |

**Supplementary table 2: Details of primary and secondary antibodies**

| Name | Dilution | Supplier | ­­Catalog no. |
| --- | --- | --- | --- |
| Bax | 1:1000 | Affinity | AF0120 |
| Bcl-2 | 1:1000 | Affinity | AF6139 |
| Cleaved Caspase3 | 1:1000 | Affinity | AF7022 |
| p-IκBα | 1:1000 | CST | 2859 |
| IκBα | 1:1000 | CST | 4812 |
| p-P65 | 1:1000 | CST | 3033 |
| P65 | 1:1000 | CST | 8242 |
| p-P38 | 1:1000 | Affinity | AF4001 |
| P38 | 1:1000 | Affinity | AF6456 |
| p-JNK | 1:1000 | Affinity | AF3318 |
| JNK | 1:1000 | Affinity | AF6318 |
| p-ERK | 1:1000 | Affinity | AF1015 |
| ERK | 1:1000 | Affinity | AF0155 |
| GAPDH | 1:5000 | Affinity | AF-7021 |
| HRP-Goat anti-rabbit IgG (H+L) | 1:5000 | Affinity | S001 |


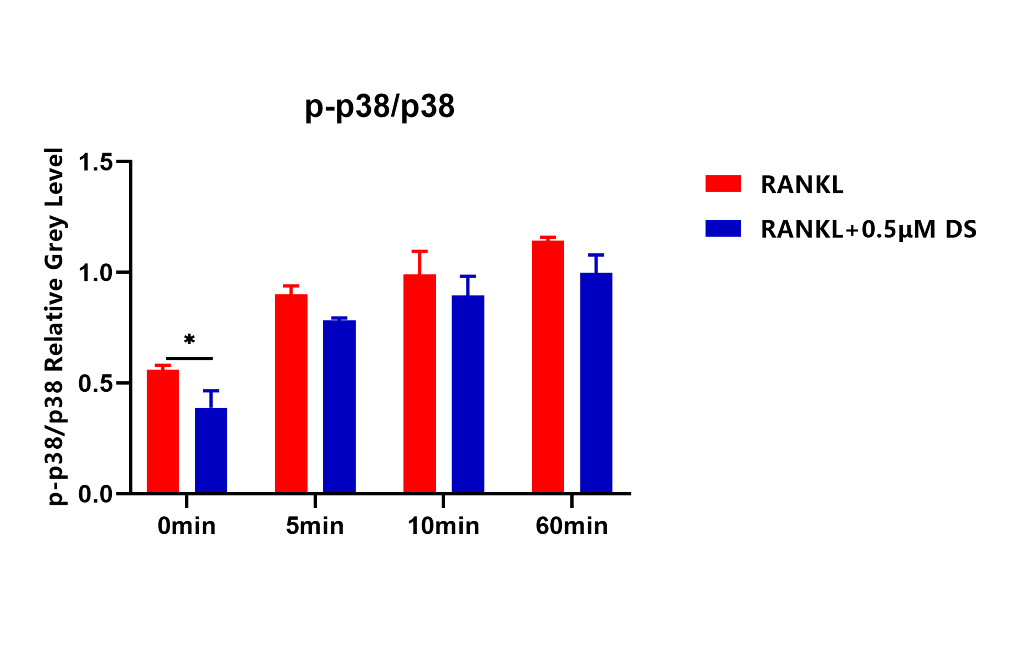
**Supplementary Figure 1：**Quantification of p38 activation as the ratio between phosphorylated and non-phosphorylated forms of proteins

**
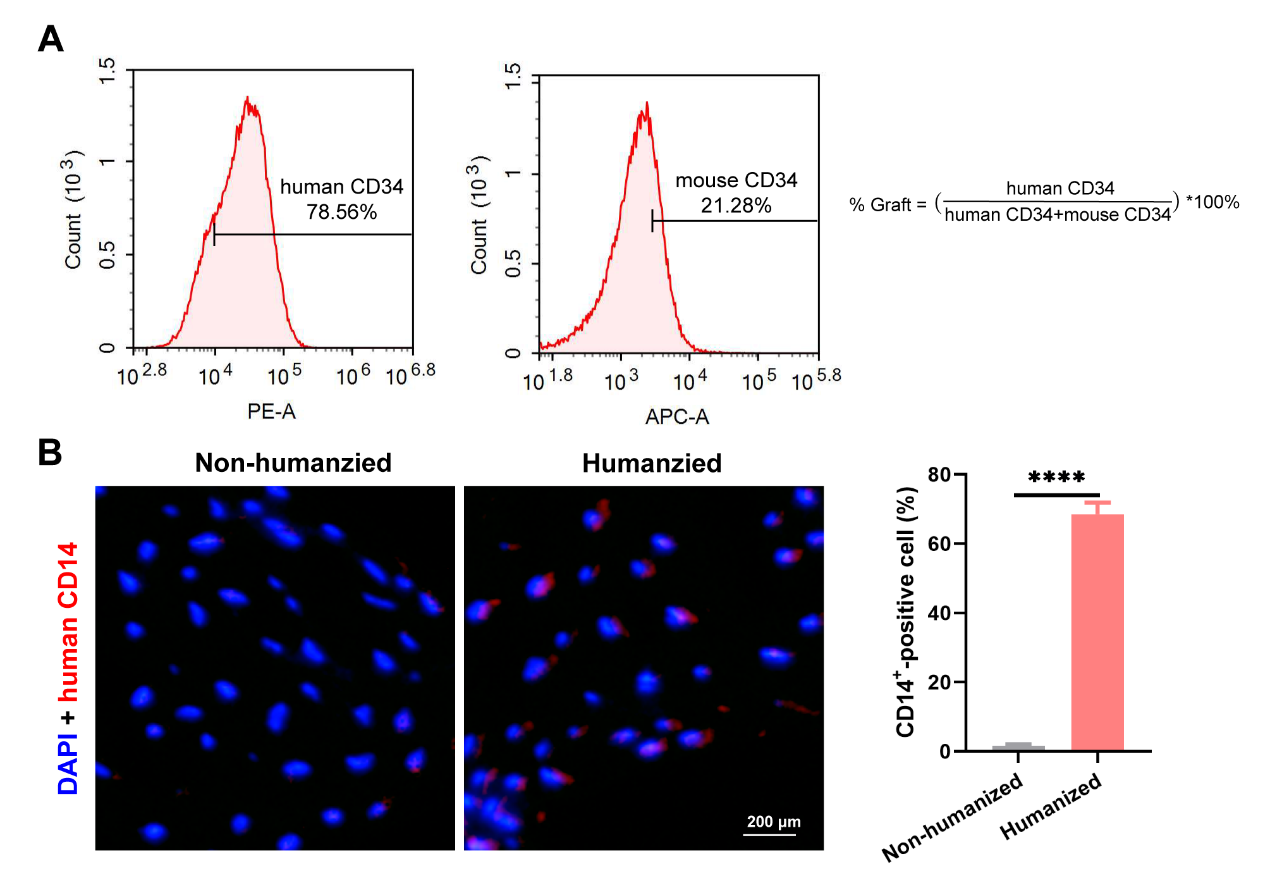
**

**Supplementary Figure 2：**Validation of the mouse humanization. (A) The flow cytometry analysis was used to calculate engraftment efficiency. % Graft was calculated as the proportion of human CD45^+^ cells in the total CD45^+^ cell population (human and mouse) in peripheral blood. (B) Immunofluorescence staining and quantification for human CD14 in the spleens of the mice.
